# Dissecting *in vivo* responses of phytohormones to *Alternaria solani* infection reveals orchestration of JA- and ABA-mediated antifungal defenses in potato

**DOI:** 10.1093/hr/uhac188

**Published:** 2022-08-25

**Authors:** Lijia Zheng, Pan Yang, Zijian Niu, Mengjun Tian, Jinhui Wang, Chaofei Sun, Shuo Zhang, Zechi Peng, Jiehua Zhu, Zhihui Yang

**Affiliations:** Hebei Agricultural University, College of Plant Protection, Baoding, Hebei, China, 071000; Hebei Agricultural University, College of Plant Protection, Baoding, Hebei, China, 071000; Hebei Agricultural University, College of Plant Protection, Baoding, Hebei, China, 071000; Hebei Agricultural University, College of Plant Protection, Baoding, Hebei, China, 071000; Hebei Agricultural University, College of Plant Protection, Baoding, Hebei, China, 071000; Hebei Agricultural University, College of Plant Protection, Baoding, Hebei, China, 071000; Hebei Agricultural University, College of Plant Protection, Baoding, Hebei, China, 071000; Hebei Agricultural University, College of Plant Protection, Baoding, Hebei, China, 071000

Dear Editor,

Phytohormones play vital roles in plant survival under incessant abiotic and biotic stresses. On perception of a pathogen invasion, plants quickly activate a complex network of phytohormone signals to defend against it [1]. The general opinion is that salicylic acid (SA) mediates plant resistance to biotrophic and hemi-biotrophic pathogens and jasmonic acid (JA) acts against necrotroph [2, 3]. A recent report showed that SA but not JA signaling is necessary for potato defense against *Alternaria solani* (*A. solani*), a necrotrophic pathogen causing leaf chlorosis and tissue necrosis [4]. This suggested that JA is ineffective during *A. solani* infection. In addition to SA, crosstalk between JA and other phytohormones has been reported widely. Thus, the function of the dialogue between JA and other phytohormones in response to necrotroph infection should be re-evaluated in potato plants. However, there is a lack of understanding about the *in vivo* responses of JA and other phytohormones. Here, we report the antagonistic roles of JA and abscisic acid (ABA) in response to *A. solani* infection in potato plants.

We inoculated potato plants (cv. Favorita, a variety susceptible to *A. solani*) with *A. solani* in a greenhouse by foliar sprays with spore suspensions (1 × 10^5^/mL) of *A. solani* isolate HWC-168 [5] and found slight early blight symptoms appearing occasionally on the infected leaves at 24 hours post inoculation (hpi) (Fig. 1A, 2^nd^ panel). The disease symptoms developed rapidly in the subsequent 24 h, and a dozen necrotic spots were observed on leaves at 36 hpi (Fig. 1A, 3^rd^ panel), and severe necrotic spots appeared on leaves at 48 hpi (Fig. 1A, 4^th^ panel). To quantify the chlorosis degree of leaves, we measured the contents of chlorophylls (chlorophyll a and b) by using leaves post 24, 36, and 48 hpi. The chlorophyll contents in the infected leaves decreased persistently at 36 hpi and 48 hpi (Fig. 1B). Based on these symptoms and chlorophyll levels, we used 24, 36, and 48 hpi as representative time points of early-, medium-, and late-infection stages, during which the antagonistic responses were classed as resistant, hindered, and failed, respectively.

**Figure 1:**
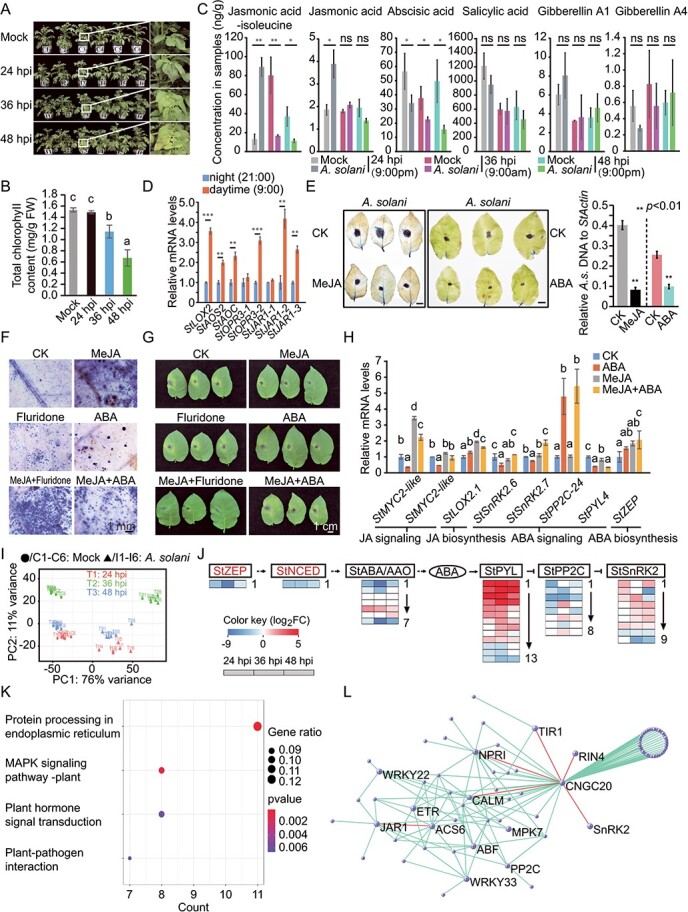
Complex crosstalk of JA- and ABA-mediated antifungal defense in potato plants. A. Disease symptoms of early blight in leaves of potato plants that developed after being inoculated with *A. solani*. B. Total chlorophyll contents of potato leaves were significantly decreased at 36 and 48 hpi. Bars with different letters indicate significant differences between groups (p < 0.01, by Welch’s ANOVA followed by the Games-Howell test), and error bars indicate 95% confidence interval. FW, fresh weight. C. Plant hormone levels in potato leaves of mock-inoculated (Mock) and *A. solani*-infected (*A. solani*) plants. Student’s *t* test: ^*^p < 0.05, ^**^p < 0.01 and ^***^p < 0.001; ns represents no significant. Bars represent standard errors. Each treatment was conducted with three biological repeats. The sampling times and temperatures at 24, 36, and 48 hpi were 21:00/20°C, 9:00/22°C, and 21:00/20°C, respectively. D. qPCR was used to verify the expression of eight genes responsible for JA biosynthesis. The samples were collected at 9:00/20°C (daytime) and 21:00/22°C (night); Student’s *t* test: ^*^p < 0.05, ^**^p < 0.01 and ^***^p < 0.001; Vertical bars indicate the standard deviations of three biological replicates. E. Trypan blue staining of *A. solani* infected leaves (72 hpi) pre-treated (24 h) with MeJA (left panel, 10 μM) or ABA (middle panel, 100 μM). CK, control check. *A. solani* biomasses in each sample were quantified by qPCR (right panels). F and G. NBT staining and resistance identification of potato leaves pre-treated (24 h) with MeJA (10 μM) or ABA (100 μM), and Fluridone (10 μM) separately or coordinately; each treatment had three replicates times. Bar = 1 mm. H. Detection of expression levels of selected genes from JA and ABA synthesis and signaling pathways. Bars with different letters indicate standard errors (see B for the methods). Vertical bars, indicate standard errors. I. Principal component analysis (PCA) of normalized RNA-Seq read counts after variance stabilizing transformation in the DESeq2 database. Sample to sample distances are illustrated on the first two principal components (PCs) comprising approximately 88% of the variation. Six biological replicates were performed. J. Gene expression changes in the ABA response pathway after inoculated with *A. solani*. The heat map shows the changes in gene expression. FC, fold change. K. KEGG analysis of “salmon” module genes related to phytohormone content levels. L. Phytohormone-related “samonl” module, the enlarged circle represents the hub gene encoded according to its biological function, and correlations between the hub genes are indicated by red connecting lines. *WRKY33*, *CNGC20*, *RIN4*, *ACS6,* and *CALM* are plant-pathogen interaction related genes. *PP2C, SnRK2, ERS, TIR1, JAR1, ABF,* and *NPR1* are phytohormone transduction related genes. All the gene IDs in this study were listed in Table 1.

**Table 1:** List of gene name/ID used in the study

**Gene Name**	**Gene ID**	**Gene Name**	**Gene ID**	**Gene Name**	**Gene ID**	**Gene Name**	**Gene ID**
Figure 1D		Figure 1J		StPYL 11	LOC102604335	Figure 1L	
StLOX2.1	LOC102596122	StZEP 1	LOC102592281	StPYL 12	LOC102591194	WRKY33	LOC102588093
StAOS2	LOC102589003	StNCED 1	LOC102577783	StPYL 13	LOC102596997	WRKY22	LOC102584992
StAOC	LOC102577822	StABA/AAO 1	LOC102584670	StPP2C 1	LOC102596252	TIR1	LOC102595865
StOPR3–1	LOC102586986	StABA/AAO 2	LOC102577811	StPP2C 2	LOC102593313	SnRK2	LOC102599466
StOPR3–2	LOC102581161	StABA/AAO 3	LOC102580056	StPP2C 3	LOC102601835	RIN4	LOC102596147
StJAR1–1	LOC102600861	StABA/AAO 4	LOC102501215	StPP2C 4	LOC102599348	PP2C	LOC102599348
StJAR1–2	LOC102603612	StABA/AAO 5	LOC102588119	StPP2C 5	LOC102582408	NPRI	LOC102592156
StJAR1–3	LOC102578750	StABA/AAO 6	LOC102598224	StPP2C 6	LOC102591856	MPK7	LOC102592852
		StABA/AAO 7	LOC102599541	StPP2C 7	LOC102582883	JAR1	LOC102603612
Figure 1H		StPYL 1	LOC102578907	StPP2C 8	LOC102579707	ETR	LOC102577920
StMYC2-like	LOC102590233	StPYL 2	LOC102602722	StSnRK2 1	LOC102580461	CNGC20	LOC102579401
StMYC2-like	LOC102597494	StPYL 3	LOC102580652	StSnRK2 2	LOC102599466	CALM	LOC102593468
StLOX2.1	LOC102596122	StPYL 4	LOC102591866	StSnRK2 3	LOC102592497	ACS6	LOC102577619
StSnRK2.6	LOC102599466	StPYL 5	LOC102580567	StSnRK2 4	LOC102587696	ABF	LOC102585425
StSnRK2.7	LOC102580461	StPYL 6	LOC102594622	StSnRK2 5	LOC102582360		
StPP2C-24	LOC102601835	StPYL 7	LOC102604114	StSnRK2 6	LOC102600490		
StPYL4	LOC102591866	StPYL 8	LOC102580526	StSnRK2 7	LOC102589067		
StZEP	LOC102592281	StPYL 9	LOC102578373	StSnRK2 8	LOC102599363		
		StPYL 10	LOC102584412	StSnRK2 9	LOC102590012		

Mock-inoculated (Mock) and *A. solani-*inoculated potato leaves at 24, 36, and 48 hpi were used for plant hormone measurement. Compared with mock samples, the levels of bioactive jasmonoyl-isoleucine (JA-Ile) were 6.7 times at 24 hpi but 0.2 times and 0.3 times at 36 hpi and 48 hpi, respectively (Fig. 1C). We noted that the JA-Ile level increased dramatically in mock at 36 hpi compared with those in other mock samples (Fig. 1C), possibly due to the expression levels of JA biosynthesis genes are higher in the daytime than at night (Fig. 1D). This result is consistent with the regulation of the plant immune response by the circadian clock in *Arabidopsis* [6]. A significant decrease in ABA content was triggered by *A. solani* infection while neither SA nor gibberellins (GAs) contents were changed markedly at three infection time points (Fig. 1C). To clarify the roles of JA and ABA in resisting *A. solani*, we treated potato leaves with exogenous methyl jasmonate (MeJA) or ABA, respectively, and then inoculated with *A. solani*. The lesions and *A. solani* biomasses of MeJA- or ABA- treated leaves were much smaller and less than in control treatment leaves (Fig. 1E), revealing the effects of JA and ABA on the resistance of potato to *A. solani* in potato.

To identify the reasons of decrease of ABA contents in potato leaves infected by *A. solani*, we performed mixed treatments of MeJA, ABA, and the ABA biosynthesis inhibitor (Fluridone) to the surface of potato leaves, and then tested the levels of reactive oxygen species (ROS) by nitro blue tetrazolium (NBT) staining, and estimated the early blight disease symptoms of each sample. Through analyzing the results of NBT staining and resistance identification, we surprisingly found that the combined MeJA/ABA treatment induced ROS accumulation but did not enhance the resistant activities of potato leaves compared with control treatment, and the responses were less than MeJA treatment alone (Fig. 1F, G). Unexpectedly, the ROS accumulation and the immune activities were more intense with combined MeJA/fluridone treatment than with MeJA or fluridone treatments (Fig. 1F, 1G). Quantitative real-time PCR (qPCR) analysis showed that the transcript levels of two *StMYC2* genes, responsible for JA signal transduction, were significantly lower following ABA treatment or MeJA/ABA co-treatment than in control or JA treatments, respectively (Fig. 1H). Thus, potato plants enhanced JA-mediated antifungal activity by repressing ABA contents at an early *A. solani* infection stage.

To uncover the mechanism of hormonal reaction to *A. solani* infection, samples used for hormonal analysis were also used for RNA sequencing (RNA-seq). Principal component analysis (PCA) of the 36 RNA-seq datasets showed that the samples were clearly separated based on their time-course infection stage (Fig. 1I). We first focused our attention on differentially expressed genes (DEGs) involving ABA biosynthesis. As shown in Figure 1J, most genes of this pathway were reduced to different degrees at all three infestation time points of *A. solani*, which could have caused the decrease in ABA content. Many genes in the ABA signaling pathway (*StSnRK2s*) as well as ABA receptor genes (*StPYLs*) were induced, and StSnRK2s inhibitor coding genes (*StPP2Cs*) were reduced to different degrees after *A. solani* infection (Fig. 1J). As the ABA signaling genes could reflect feedback regulated by ABA responsive factors [7, 8], we surmised that the changes in expression levels of genes in ABA signaling pathway under *A. solani* invasion might be a feedback response to ABA insufficiency. Consistent with this hypothesis, exogenous ABA treatment caused opposite trend of expression of genes in the ABA signaling pathway to *A. solani* invasion compared with control treatments, i.e. *StSnRK2.6/2.7* and *StPYL4* were repressed and *StPP2C-24* was induced by ABA treatment (Fig. 1H). Next, we used phytohormone content as the basis to perform weighted gene co-expression network analysis (WGCNA) on the transcriptome data. After removing genes with low expression and little overall change, 6323 DEGs were selected for WGCNA. In all, 14 gene expression modules were generated in the analysis, among which DEGs identified by the salmon workflow (module) were significantly related to plant hormones. Further analysis in the kyoto encyclopedia of genes and genomes (KEGG) database revealed that DEGs in the salmon module were enriched in four types of biological activities, including protein processing, mitogen-activated protein kinase (MAPK) signaling, plant hormone signal transduction and plant-pathogen interaction (Fig. 1K). Among the DEGs of the salmon module, genes involved in plant-pathogen interaction and hormone signal transduction appeared as hubs in the network (Fig. 1L). In particular, *StCNGC20*, encoding a membrane channel protein that controls Ca^2+^ influx [9], was associated with many genes in the module, suggesting that it might play important regulatory roles in the phytohormone-mediated plant immune response (Fig. 1L) [10].

Individual MeJA or ABA treatment enhanced plant resistance, but the activities of MeJA/ABA co-treatment were less efficient than of MeJA or ABA single treatments (Fig. 1F, G), so the plants activated only one of the two hormone pathways against *A. solani*. The effect of JA was better than ABA (Fig. 1E, right panel); moreover, inhibiting ABA biosynthesis enhanced the antifungal activities of JA (Fig. 1F, G), when encountering *A. solani*. Thus, the “best choice” for potato plants is to increase JA and decrease ABA contents. This work further our understanding of the complex coordination between JA and ABA in responding to pathogen infections *in vivo* and offers opportunities to discover novel strategies for improving plant immune activities.

## Acknowledgements

This work was funded by Hebei Key Research and Development Program (21326515D), China Agriculture Research System of MOF and MARA (CARS-09-P18), and The National Natural Science Foundation of China (32070143).

## Author contributions

L.J.Z. designed the experiments; L.J.Z., P.Y., Z.J.N., M.J.T., J.H.W., C.F.S., S.Z. and Z.C.P. performed the experiments; L.J.Z. wrote the manuscript. J.H.Z. and Z.H.Y. supervised the project and edited the manuscript.

## Data availability

Raw reads have been submitted as a BioProject under accession PRJNA574559.

## Conflict of interest

The authors declare no competing interests.
